# Characterization and Source Analysis of Water-Soluble Ions in PM_2.5_ at Hainan: Temporal Variation and Long-Range Transport

**DOI:** 10.3390/toxics13090804

**Published:** 2025-09-22

**Authors:** Xinghong Xu, Wenshuai Xu, Xinxin Meng, Xiaocong Cao, Biwu Chu, Chuandong Du, Rongfu Xie, Zhaohe Zeng, Hui Sheng, Youjing Lin, Weijun Yan, Hong He

**Affiliations:** 1 Hainan Provincial Ecological and Environmental Monitoring Center, Haikou 571126, China; sinhom_xu@foxmail.com (X.X.);; 2 Hainan Research Academy of Environmental Sciences, Haikou 571126, China; 3 Research Center for Eco-Environmental Sciences, Chinese Academy of Sciences, Beijing 100085, China; 4 School of Ecology, Hainan University, Haikou 570228, China

**Keywords:** regional transport, PM_2.5_, water-soluble ions, source apportionment

## Abstract

We explored the mass concentrations of water-soluble ions in PM_2.5_ and their variations across different time scales and concentration levels. Using the Positive Matrix Factorization (PMF) model and backward trajectory analysis, we focused on identifying the sources of PM_2.5_ and its water-soluble ion fractions, with particular emphasis on regional transport. The findings reveal that the average mass concentration of total water-soluble ions in Hainan between 1 August 2021 and 31 July 2022 was 7.0 ± 4.4 µg m^−3^, constituting 73.5% ± 24.4% of PM_2.5_. Secondary ions (SO_4_^2−^, NO_3_^−^, NH_4_^+^) were dominant, accounting for 84.0% ± 12.4% of the total water-soluble ions, followed by sea-salt particles. Seasonal variations were pronounced, with the highest concentrations observed in winter and the lowest in summer. The results of the PMF analysis showed that secondary sources, combustion sources, dust sources, and oceanic sources are the main sources of PM_2.5_ at the monitoring site. The potential sources and transport pathways of water-soluble ions exhibit distinct seasonal characteristics, with the land-based outflows from the YRD–PRD–Fujian corridor controlling Hainan’s PM_2.5_ maxima, while southerly marine air delivers the annual minimum; seasonal alternation between dust/secondary aerosols (winter–spring), combustion (autumn), and oceanic dilution (summer) dictates the island’s air-quality rhythm.

## 1. Introduction

As a major component of atmospheric pollutants, PM_2.5_ not only reduces atmospheric visibility and triggers hazy weather, but also significantly affects the climate [1,2,3,4]. Due to its small size, PM_2.5_ is not only capable of penetrating deep into the respiratory system, posing increased health risks [5,6,7], but is also easily influenced by atmospheric circulation, enabling it to be transported over long distances to downstream regions. In particular, fine particles can be transported from their sources to leeward regions due to the influence of the Asian Northeast Monsoon, which is prevalent in East Asia. This transport mechanism leads to elevated PM_2.5_ levels in the affected areas [8,9].

PM_2.5_ has a complex composition, including water-soluble ions (WSI), carbon components, trace elements, and crustal elements. WSI, particularly sulfate (SO_4_^2−^), nitrate (NO_3_^−^), and ammonium (NH_4_^+^) (collectively termed SNA), dominate PM_2.5_ mass, contributing 45–65% of its total composition [10]. These secondary inorganic aerosols form through complex atmospheric reactions influenced by precursor gases (SO_2_, NO_x_, and NH_3_) and meteorological conditions [11]. To date, many scholars have conducted a series of in-depth studies on the composition, existence form, and transformation characteristics of water-soluble ions in PM_2.5_. However, most of the studies focused on the relatively heavily polluted urban areas such as Beijing-Tianjin-Hebei [12,13], the Yangtze River Delta [14], and the Pearl River Delta [15]. In contrast, there is a notable lack of studies on PM_2.5_ in areas with lower concentrations, particularly regarding the transport mechanisms of PM_2.5_ in these regions.

Hainan Province, located at the southernmost tip of China, is characterized by a relative lack of large-scale industries and enterprises. This scarcity of local emission sources has historically contributed to its reputation for excellent air quality over the past decades [16]. Local pollutants have a negligible influence at Hainan, so long-range transport is the primary route for air pollutants, especially transboundary pollution via monsoonal flows and biomass-burning plumes [17,18]. According to reports, 41.6% of PM_2.5_ in Hainan originates from regional transport from mainland China and neighboring Southeast Asian countries [19]. These transboundary inputs complicate air quality management, underscoring the need for source apportionment.

Accordingly, continuous online monitoring of water-soluble ions in PM_2.5_ at the regional transport site of Hainan was carried out for one year, from August 2021 to July 2022, to analyze the temporal distribution characteristics of PM_2.5_ and its water-soluble ions. The sources of the water-soluble ions in PM_2.5_ were analyzed by combining the Positive Matrix Factorization (PMF) model and the backward trajectory model. The impacts of air masses in different directions on the air quality of the regional station during different seasons were illustrated. The findings of this research are intended to contribute to the scientific basis for the refined prevention and control of atmospheric pollution, specifically tailored to the unique environmental context of tropical islands close to the mainland.

## 2. Materials and Methods

### 2.1. Monitoring Locations and Monitoring Time

**Figure 1:**
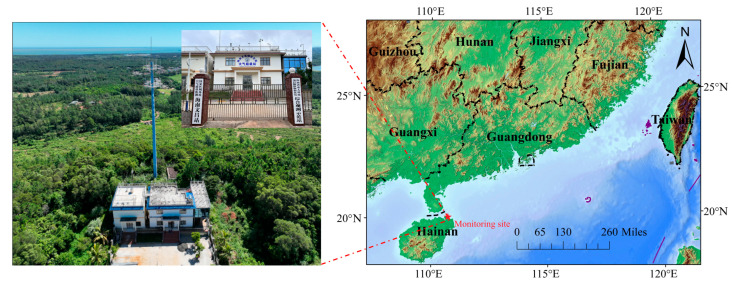
The location of the regional station of Hainan (https://www.91weitu.com/, accessed on 31 August 2025).

All data in this study were monitored using online monitoring at the Hainan Wenchang Puqian Regional Atmospheric Super Station (hereafter referred to as the “Regional station”) located at the top of Daling Mountain (20°05′ N, 110°36′ E), which is the regional transport point for Hainan Province. The location is depicted in Figure 1. Surrounded by the sea on three sides, with no obvious pollution sources, and high vegetation cover and broad-leaved forests, the regional station is an important station on the atmospheric transport corridor from the Guangdong–Hong Kong–Macao Greater Bay Area to the Hainan Free Trade Port. The monitoring period for this study was from 1 August 2021 to 31 July 2022 at hourly intervals.

### 2.2. Monitoring Instruments

PM_2.5_ samples were monitored using the Thermo Fisher 5030i instrument by Thermo Fisher Scientific Inc., Franklin, MA, USA. The equipment used for monitoring water-soluble ions was the gas–aerosol water-soluble ion online analysis system (MARGA/ADI2080) by Metrohm, Herisau, Switzerland, and a PM_2.5_ cyclone was employed to measure the mass concentration of water-soluble inorganic ions (Cl^−^, NO_3_^−^, SO_4_^2−^, Na^+^, K^+^, NH_4_^+^, Mg^2+^, and Ca^2+^) in the aerosol phase at a time resolution of one hour. The system includes a sampling system, an analysis system, a control system, and an auxiliary system. The sampling system utilizes the different diffusion properties of gas and aerosol. Soluble gas is quantitatively absorbed by the rotary liquid cavitator (WRD), while aerosol is collected by the vapor jet aerosol collector (SJAC) through the WRD without any loss. The liquid stream from the WRD and SJAC is collected by the burette, degassed, mixed with the internal standard, and then injected quantitatively into anion and cation chromatography for analysis. The analytical system is a dual-channel ion chromatography system with two built-in independent analytical modules, one for anion and organic acid analysis and one for cation analysis. The Cl^−^, NO_3_^−^, SO_4_^2−^, Na^+^, K^+^, NH_4_^+^, Mg^2+^, and Ca^2+^ detection limits were 0.01, 0.05, 0.04, 0.05, 0.09, 0.05, 0.06, and 0.09 μg m^−3^, respectively.

Quality assurance and quality control of the MARGA system include the following steps. Firstly, the inlets and sampling lines were cleaned weekly with ultrapure deionized water and dried with zero-grade air. Secondly, a primary standard flowmeter traceable to the National Institute of Metrology, China, was used to measure and verify the atmospheric inlet flow rate every month. Third, a liquid blank was obtained by operating the MARGA with the air pumps disconnected and the denuder inlets sealed, so that only the absorption solution was sampled. With the air pumps off and the denuder inlets sealed, an external-standard test was conducted by substituting the absorption solution with a known liquid standard containing SO_4_^2−^, NH_4_^+^, and NO_3_^−^, thereby verifying the analytical accuracy established by the internal lithium bromide (LiBr) standard.

### 2.3. Methods of Analysis

#### 2.3.1. Positive Definite Matrix Factorization (PMF)

The positive definite matrix factorization (PMF) model was developed by the U.S. Environmental Protection Agency (EPA) and has been widely used for PM_2.5_ component source resolution [20]. In this study, PMF 5.0 software was used for PM_2.5_ source resolution, the basic principle of which is to calculate the error of each chemical component in particulate matter using weights, and then determine its main pollution sources and contribution by the least squares method [21], with the following formula:

(1) $$X_{ij}=\sum_{k=1}^{p}g_{ik}f_{kj}+e_{ij}$$

where *X_ij_* is the concentration of the *ij*th sample, *g_ik_* denotes the contribution of the *ik*th sample, *f_kj_* denotes the mass fraction of the *kj*th sample, and *e_ij_* denotes the residual between the measured mass concentration of the *ij*th sample and its analyzed value:

(2) $$Q_{ij}=\sum_{i=1}^{n}\sum_{j=1}^{m}\left[\frac{X_{ij}-\sum_{k=1}^{p}g_{ik}f_{kj}}{u_{ij}}\right]$$

where *Q_ij_* is the sum of all sample residuals and their uncertainty *u*:

(3) $$u=\frac{5}{6}\times MDL$$

$$u=\sqrt{{{\left(error\;and\;fraction\;\times \;concentration\right)}^{2}+(0.5\times MDL)}^{2}}$$

where *u* is the uncertainty. *MDL* is the method detection limit. If the concentration is less than or equal to the *MDL* provided, Unc is calculated using a fixed fraction of the *MDL*. If the concentration is greater than the *MDL*, the calculation is based on the concentration fraction and *MDL*.

The principles of PMF have been described in detail in the PMF 5.0 User Guide (USEPA, 2014). In total, eight chemical components were used for the PMF model, which included Cl^−^, NO_3_^−^, SO_4_^2−^, Na^+^, K^+^, NH_4_^+^, Mg^2+^, and Ca^2+^. First, we calculated the uncertainty (*u*) for each species based on its concentration fraction and *MDL*. Second, different numbers of factors were tested with random seeds in 100 iterations of each run. When four factors were specified, with most species having a correlation coefficient above 0.85, the species followed a normal distribution (Table 1). Third, bootstrap (BS), displacement (DISP), and bootstrapping with displacement (BS-DISP) diagnostics were applied to assess the reliability of the resolved factor profiles and contributions. BS detects and quantifies the undue influence of a small subset of observations on the solution and, to a lesser extent, rotational ambiguity. The value of the f peak strength was ensured to be 0.5 to eliminate the rotation ambiguity. BS mapping exceeded 90% for every factor, and there were no swaps with DISP and BS-DISP. Therefore, it is suggested that four factors were appropriate in this study.

#### 2.3.2. HYSPLIT Model

The Hybrid Single-Particle Lagrangian Integrated Trajectory (HYSPLIT) model was first developed by the National Oceanic and Atmospheric Administration of the United States of America and the Australian Bureau of Meteorology to analyze pollutant sources, transport, and dispersion trajectories in conjunction with air-mass trajectories and monitoring data [22]. The model system is a hybrid Lagrangian and Eulerian diffusion model, which uses the Lagrangian method to deal with advection and diffusion processes, while the Eulerian method is used to calculate the pollutant concentrations [23].

To investigate the pollution characteristics and sources of PM_2.5_ and its water-soluble ionic constituents along different air-mass trajectories, we simulated backward trajectories with the TrajStat plug-in (developed from the HYSPLIT model) in MeteoInfo 3.5.10. The Global Data Assimilation System (GDAS) meteorological fields at 1° × 1° resolution were used, with a starting height of 500 m, a backward scale of 72 h, and a temporal resolution of 1 h. Representative months—January, April, July, and October—were selected to capture the four seasons during the monitoring period. A K-means clustering algorithm was then applied to classify the trajectories into distinct clusters. Due to the fact that a single backward trajectory is highly uncertain, we computed multiple trajectories and subjected them to cluster analysis. This multivariate statistical technique, based on the angle-distance algorithm, quantifies the pollution–concentration relationships within each source region.

## 3. Results and Discussion

### 3.1. Pollution Characteristics of PM_2.5_ and Water-Soluble Ions

#### 3.1.1. Characteristics of Seasonal Changes

**Figure 2:**
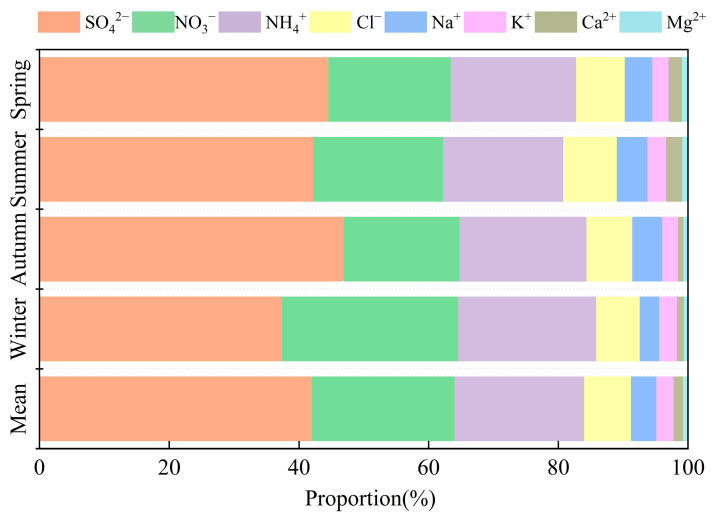
Seasonal proportion of water-soluble inorganic ions.

During the monitoring period, the mean mass concentration of PM_2.5_ at the regional station was 9.5 µg m^−3^, in which all the daily concentration data were lower than the average daily Class I standard for air quality in China (35 µg m^−3^), which shows that the study area belongs to a cleaner area. The concentrations of the eight water-soluble ions are shown in Table 2, and the proportion of each water-soluble ion to the total water-soluble ions (TWSI) is shown in Figure 2. The annual average concentration of TWSI in PM_2.5_ was 7.0 µg m^−3^, which accounted for 73.5% of the mass concentration of PM_2.5_, indicating that water-soluble ions are an important component of PM_2.5_. The mass concentrations of the eight water-soluble ions were in the following order: SO_4_^2−^ > NO_3_^−^ > NH_4_^+^ > Cl^−^ > Na^+^ > K^+^ > Ca^2+^ > Mg^2+^, of which the mass concentrations of SO_4_^2−^, NO_3_^−^, and NH_4_^+^ were 2.9 µg m^−3^, 1.5 µg m^−3^, 1.4 µg m^−3^, accounting for 41.4%, 21.4%, and 20.0%, and totaling 82.8%, of the TWSI. These were higher than those in Beijing (75.2%) [24], Nanjing (76.8%) [25], and Sanya (57.8%) [26], reflecting that the study area had a strong secondary pollution characteristic. In addition to SNA, the mass concentrations of sea-salt ions such as Cl^−^ and Na+ totaled 0.8 µg m^−3^, accounting for 11.4% of the TWSI, which is significantly higher than that of non-coastal cities such as Beijing and Nanjing [24,25], and is clearly influenced by the ocean.

In this study, March–May 2022 was defined as spring, August 2021 and June–July 2022 as summer, September–November 2021 as autumn, and December 2021–February 2022 as winter. The mass concentrations of TWSI during the monitoring period showed a clear seasonal variation, from highest to lowest: winter (10.4 µg m^−3^) > spring (7.4 µg m^−3^) > autumn (6.3 µg m^−3^) > summer (3.9 µg m^−3^), with the highest concentration season (winter) being 2.67 times higher than the lowest season (summer). The seasonal variations in SNA are influenced by different sources of ions, meteorological factors, and chemical reaction conditions [27]. The mass concentrations of SNA are consistent with a change in TWSI concentrations, and the proportions of SNA in TWSI (80.8–85.9%) were relatively stable across seasons, indicating that SNA is the main driver of TWSI concentration changes. The higher concentrations of SO_4_^2−^, NO_3_^−^, and NH_4_^+^ in winter than in summer could be attributed to the following: (1) the lower temperature and higher humidity in winter, which favors the conversion of NH_3_ and HNO_3_ to particulate states (NH_4_)_2_SO_4_ and NH_4_NO_3_; (2) the higher temperatures and more frequent precipitation in summer, which reduces SO_4_^2−^, NO_3_^−^, and NH_4_^+^ concentrations [28]; and (3) the influences from anthropogenic emissions and regional transportations, which will be discussed in later sections. Among the other ions, the concentrations of Na^+^ and Mg^2+^ were the highest in spring and were contributed by surrounding marine sources. Ca^2+^, as an important tracer of soil and sand and dust sources [29], showed high concentrations in spring and winter, which should be explained by the concurrent high-speed northeasterly winds and low precipitation. The concentrations of Cl^−^ and K^+^ were the highest in winter and the lowest in summer, with different seasonal variation characteristics from those of Na^+^ and Mg^2+^ (highest in spring and lowest in summer), suggesting that Cl^−^ is not only affected by marine sources. The unfavorable meteorological conditions and the increase in biomass burning might have led to the increase in the concentrations of K^+^ and Cl^−^ [30].

#### 3.1.2. Characterization of Daily Changes

**Figure 3:**
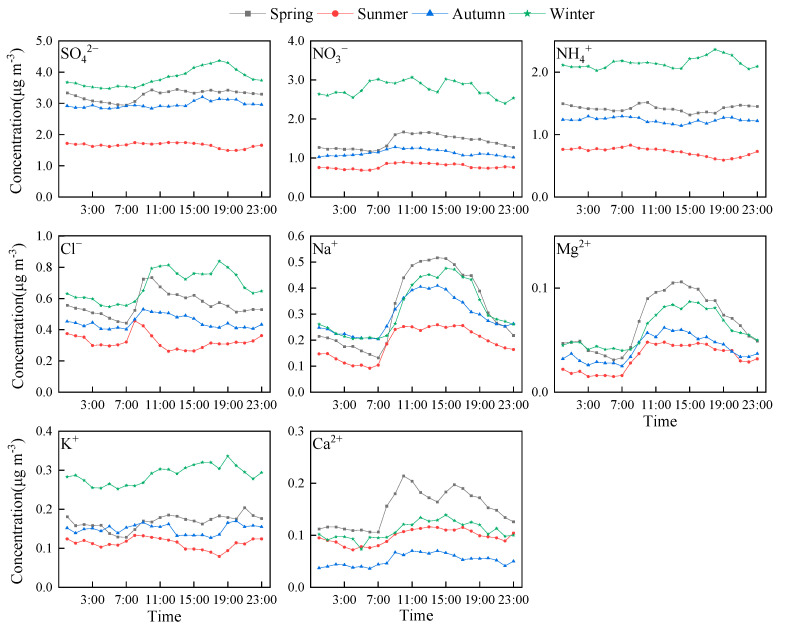
Diurnal variation characteristics of water-soluble inorganic ions in different seasons.

Figure 3 presents a detailed analysis of the daily fluctuation patterns of water-soluble ions throughout different seasons. The SNA had little fluctuation of the daily change in spring, summer, and autumn, but in winter, when SNA concentrations were high, daily changes were more pronounced. SO_4_^2−^ and NH_4_^+^ showed a single-peak pattern, peaking around 6:00 PM, while NO_3_^−^ showed a double-peak pattern, peaking around 11:00 AM and 3:00 PM. Na^+^, Mg^2+^, and Cl^−^ display a single-peak daily pattern influenced by marine sources in spring, summer, and autumn. The maximum and minimum values of Na^+^ and Mg^2+^ appeared at the same time, but there was a significant fluctuation of Cl^−^ in the evening of winter, which may be related to the biomass burning and unfavorable diffusion conditions in winter. K^+^ concentrations fluctuate throughout the day, with winter levels significantly higher than other seasons, peaking mainly in the evening and night-time. Ca^2+^ shows minor daily variations during summer, autumn, and winter, with spring experiencing greater amplitude in daily fluctuations and high concentrations concentrated between 8:00 AM and 7:00 PM. This analysis underscores the distinct daily variation patterns among water-soluble ions, indicating that their primary influencing factors differ significantly.

#### 3.1.3. Compositional Characteristics at Different PM_2.5_ Concentrations

**Figure 4:**
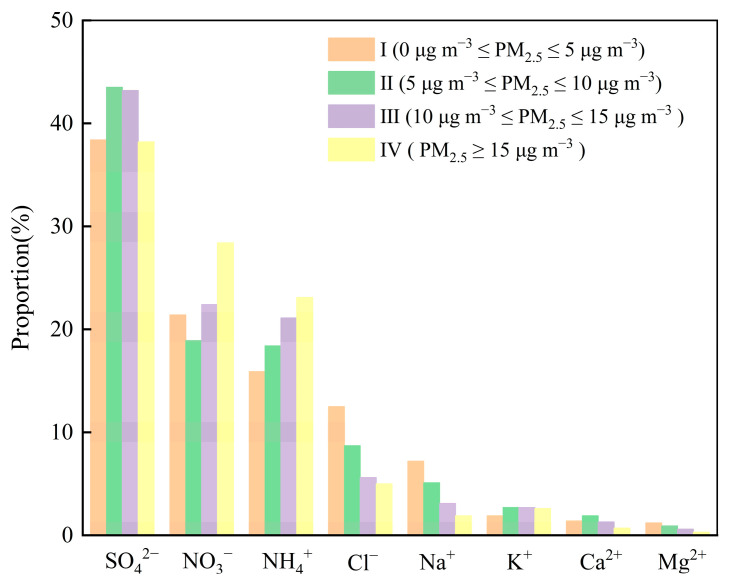
Contribution proportion of PM_2.5_ components under different pollution levels.

With the change of PM_2.5_ concentration, its composition will also change [31]. Therefore, analyzing the component characteristics of water-soluble ions at different PM_2.5_ concentrations in low-concentration areas is of great significance for the future precision control of the atmosphere at low PM_2.5_ concentrations in China. In this study, according to the World Health Organization 2021 Air Quality Guidelines as the basis of division, the concentration of PM_2.5_ was divided into four value domains: I (0 µg m^−3^ < *ρ*(PM_2.5_) ≤ 5 µg m^−3^), II (5 µg m^−3^ < *ρ*(PM_2.5_) ≤ 10 µg m^−3^), III (10 µg m^−3^ < *ρ*(PM_2.5_) ≤ 15 µg m^−3^), and IV (*ρ*(PM_2.5_) ≥ 15 µg m^−3^) are discussed. Figure 4 illustrates the proportion of each water-soluble ion in TWSI at varying PM_2.5_ concentrations. As pollution intensifies, the percentages of Na^+^, Mg^2+^, and Cl^−^ decrease, suggesting a significant marine influence on PM_2.5_ when concentrations are low. Conversely, the respective percentages of NH_4_^+^ and NO_3_^−^ across the four domains increase from 15.9% to 23.1%, and from 21.4% to 28.4%, indicating that nitrogen pollution plays a crucial role in the increase in PM_2.5_ concentration. SO_4_^2−^ consistently represents the highest proportion of water-soluble ions across different seasons and pollution levels. The monitoring site is situated in a regional background area of Hainan Province, characterized by minimal coal combustion emissions. Aside from transportation of emissions from coal combustion, the high proportion of SO_4_^2−^ may also originate from natural sources such as dimethylsulfide (DMS) produced by marine phytoplankton [32].

### 3.2. Secondary Conversion Characteristics

Sulfur oxidation rate (SOR) and nitrogen oxidation rate (NOR) can effectively reflect the extent to which gaseous precursors SO_2_ and NO_2_ are converted into secondary components SO_4_^2−^ and NO_3_^−^ [33], calculated as follow: SOR = *n*(SO_4_^2−^)/(*n*(SO_2_) + *n*(SO_4_^2−^)) and NOR = *n*(NO_3_^−^)/(*n*(NO_2_) + *n*(NO_3_^−^)).

**Figure 5:**
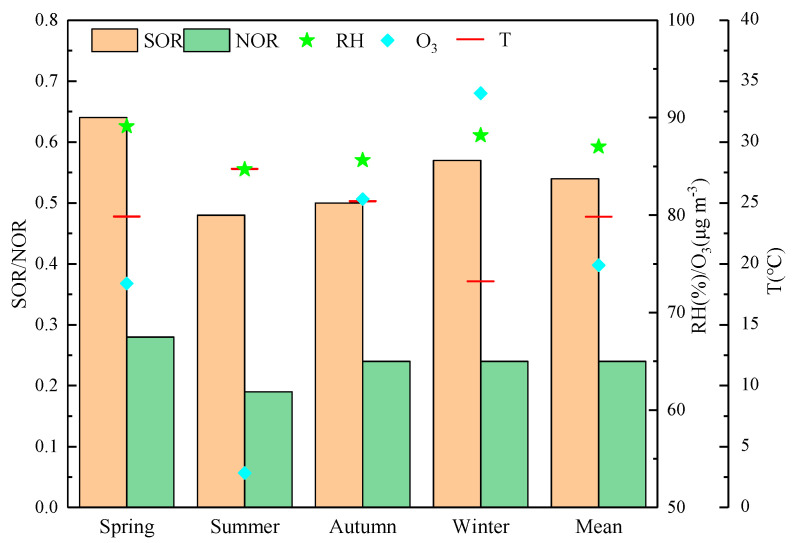
Seasonal distribution of SOR, NOR, O_3_, Relative Humidity, and Temperature. Note: “RH” means Relative Humidity, “T” means Temperature.

When SOR and NOR are less than 0.10, it indicates that the atmosphere is primarily composed of primary pollutants. In this study, as shown in Figure 5, both the SOR and NOR values are significantly greater than 0.1, with annual average values of 0.54 and 0.24, respectively. This indicates that secondary transformation reactions at the monitoring site were intense throughout the observation period. The monitoring site is located in a coastal area, where high salinity and humidity significantly promote the secondary transformation of SO_2_ and NO_2_, particularly enhancing the formation of SO_4_^2−^ [34].

The transformation of SO_4_^2−^ primarily involves gas-phase and aqueous-phase oxidation. Gas-phase oxidation occurs through the reaction of SO_2_ with atmospheric O_3_ and OH radicals, which is significantly correlated with temperature and O_3_ concentration. Aqueous-phase oxidation of SO_2_ with oxidants such as HONO, H_2_O_2_, NO_2_, and O_2_ (catalyzed by Mn and Fe) on the surfaces of water droplets and aerosols, on the other hand, is highly correlated with atmospheric humidity [35]. As depicted in Figure 5, the SOR values are higher in spring and winter compared to summer and autumn, indicating that higher humidity effectively promotes the oxidation of SO_2_. NOR were 0.28, 0.19, 0.24, and 0.24 in spring, summer, autumn, and winter, respectively, which showed little seasonal difference, except in summer. This is related to the formation mechanism of NO_3_^−^. Higher temperatures are not conducive to the reaction of NH_3_ with HNO_3_ to form particulate NH_4_NO_3_ and reduce its stability [36]. During summer, the higher temperatures could accelerate the dissociation of ammonium nitrate particles to gas-phase ammonia and nitric acid.

**Figure 6:**
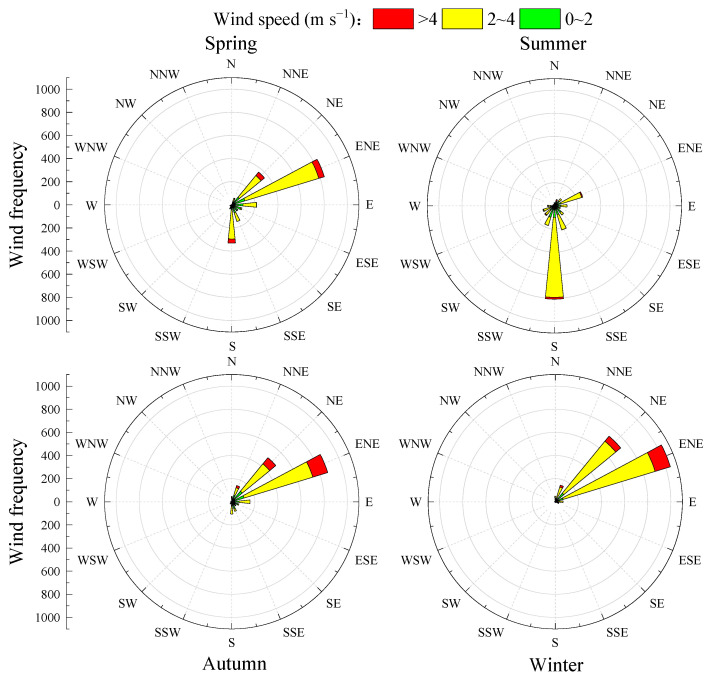
Wind rose in different seasons.

Even though elevated SOR and NOR values may not fully indicate the extent of precursor conversion in Hainan, this is due to the influence of regional transport on the urban concentrations of SO_4_^2−^ and NO_3_^−^. As depicted in Figure 6, the wind rosette for the monitoring site varies with the seasons. During summer, the prevailing winds are southerly, with the southern aspect of the site facing the South China Sea. The influx of clean maritime air currents aids in the dilution of pollutants, which likely contributes to the lower ion concentrations observed in this season. In contrast, the spring, autumn, and winter seasons are characterized by predominantly northeasterly winds. These winds are significantly stronger than those in summer. The northeastern region of the monitoring site is proximal to the northern parts of China and the Pearl River Delta. This geographic positioning facilitates the transportation of pollutants from upstream areas to Hainan, potentially leading to higher ion concentrations during these seasons.

### 3.3. Correlation Analysis of PM_2.5_ with Water-Soluble Ions and Meteorological Factors

**Figure 7:**
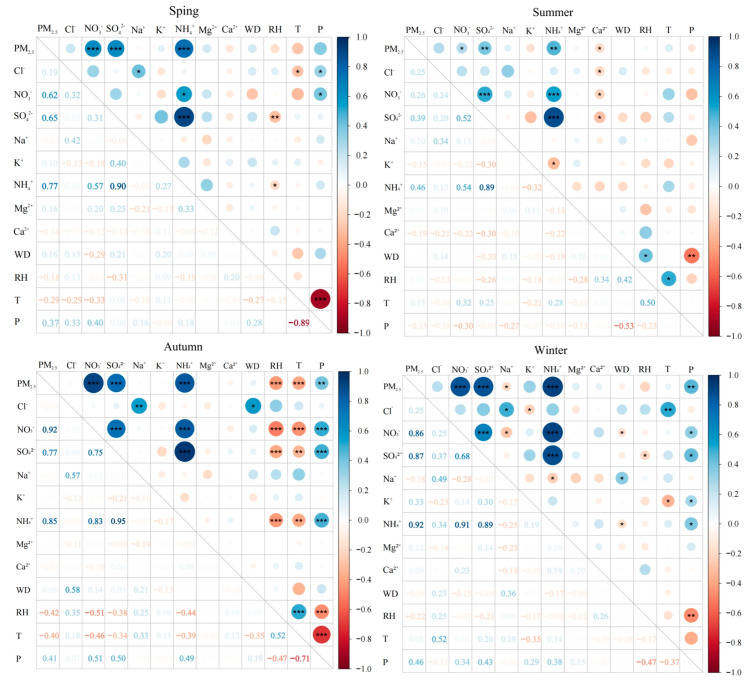
The correlation between PM_2.5_, water-soluble inorganic ions, and meteorological factors. Note: “WD” means Wind Speed, “RH” means Relative Humidity, “T” means Temperature, “P” means Barometric Pressure; *** denotes significant correlation at *p* < 0.001, ** denotes significant correlation at *p* < 0.01, and * denotes significant correlation at *p* < 0.05.

The correlation between PM_2.5_ and its water-soluble ions, as well as among the ions themselves, can shed light on their potential sources and binding mechanisms within particulate matter [37]. Figure 7 illustrates these correlations along with the influence of meteorological factors across different seasons. PM_2.5_ is strongly correlated with SNA components in spring, autumn, and winter, and shows a weaker—though still significant—correlation in summer, indicating that secondary inorganic ions make a substantial contribution to atmospheric PM_2.5_ and should not be overlooked. The high correlations among NH_4_^+^, SO_4_^2−^, and NO_3_^−^ suggest that NH_4_^+^ is probably present as (NH_4_)_2_SO_4_ and NH_4_NO_3_. In addition, the high correlation between Na^+^ and Cl^−^ indicates that these ions share a common origin; given that the site is surrounded by the sea on three sides, marine sources exert a considerable influence on PM_2.5_ composition.

In terms of meteorological influences, the correlation between PM_2.5_ and its water-soluble ion concentrations with meteorological parameters was strongest in autumn, indicating that the monitoring sites are especially sensitive to weather variability during this season. The effects of seasonal change and meteorological factors on ion concentrations also differ. In autumn, SO_4_^2−^, NO_3_^−^, and NH_4_^+^ were positively correlated with barometric pressure and negatively correlated with temperature and humidity. This pattern can be ascribed to the southward advance of the continental cold high-pressure system, which transports large amounts of highly concentrated ionic species to the sites. In summer, by contrast, the concentrations of SO_4_^2−^, NO_3_^−^, and NH_4_^+^ were positively correlated with temperature, implying that elevated temperatures promote secondary PM_2.5_ formation. Additionally, Na^+^ and Cl^−^ were positively correlated with wind speed in summer, autumn, and winter, and most significantly in autumn, suggesting that stronger winds enhance the dispersion of sea-salt ions from the ocean surface.

### 3.4. Source Analysis

**Figure 8:**
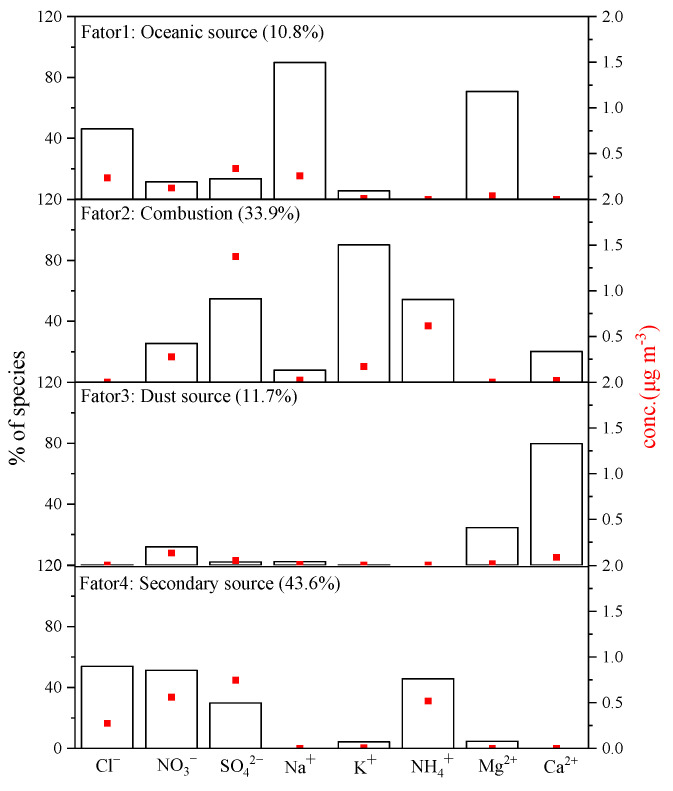
Contribution of pollution sources to water-soluble ions in PM_2.5_.

In this paper, we conducted a factor analysis of water-soluble ion fractions in PM_2.5_ during the monitoring period using the EPA PMF 5.0 software. The analysis resolved a total of four factors, and the results are presented in Figure 8. Factor 1 is characterized by high percentages of Na^+^ (89.9%), Mg^2+^ (70.8%), and Cl^−^ (46.1%). Na+ and Cl^−^ are typically indicative of sea-salt sources. The Mg^2+^/Na^+^ ratio is used to determine whether these ions in particulate matter primarily originate from the ocean or soil. A ratio less than 1.2 suggests an oceanic source [38]. The annual average Mg^2+^/Na^+^ ratio during the monitoring period was 1.1, indicating a predominant oceanic influence. Therefore, Factor 1 is identified as the oceanic source, with a contribution rate of 10.8%. Factor 2 contains relatively high percentages of K^+^ (90.1%), which is an excellent tracer of biomass-burning aerosols [39], followed by SO_4_^2−^ (54.8%), and NH_4_^+^ (54.3%). SO_4_^2−^ is formed by the photochemical oxidation of sulfur-containing precursors (SO_2_ and H_2_S) released by coal combustion and NH_4_^+^ likely derived from secondary transformations of precursors released by biomass combustion (NH_3_) [40]. Consequently, Factor 2 is recognized as a combustion source, with a contribution rate of 33.9%. Factor 3 has a high percentage of Ca^2+^ (79.8%) and Mg^2+^ (24.5%), which are typically associated with building construction, soil and road dust, and long-distance transported sand and dust [41]. Therefore, Factor 3 is identified as a dust source, with a contribution rate of 11.7%. Factor 4 features high percentages of Cl^−^ (53.8%), NO_3_^−^ (51.3%), NH4^+^ (45.7%), and SO_4_^2−^ (29.8%), which are significant characteristics of secondary sources. NO_3_^−^ and SO_4_^2−^ are mainly from the oxidation of NO_x_ and SO_2_, while NH_4_^+^ probably comes from the conversion processes between ammonia and sulfuric and nitric acid [23]. Thus, Factor 4 is identified as a secondary source, with a contribution rate of 43.6%.

### 3.5. Backward Trajectory Analysis

**Figure 9:**
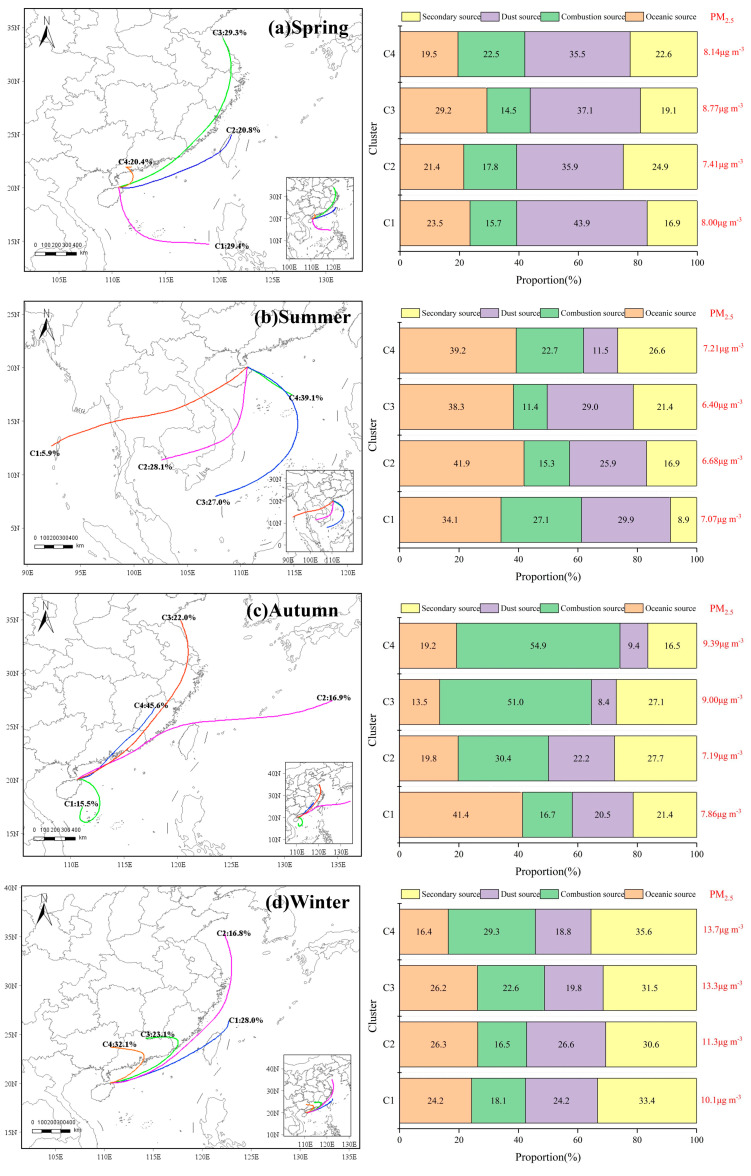
Results for the back-trajectory cluster.

Regional transport of atmospheric pollutants significantly contributes to local pollutant concentrations, particularly in areas with inherently low pollutant levels [42]. This study analyzed the pollution trajectories of different air masses in four seasons, as shown in Figure 9. Each backward trajectory in Figure 9 indicates a 72 h period transportation clustering result, using the regional background site as the simulation starting point. The clustering results show distinct seasonal and spatial differences in air-mass transport paths and result in different characteristics of the pollutants.

In spring (Figure 9a), 70.5% of the trajectories arriving at the sampling site originated from the northeast of Hainan Island; of these, 29.3% (C3) traveled over the Yangtze River Delta (YRD) and crossed Fujian Province before reaching the receptor, leading to the highest PM_2.5_ and chemical component concentrations under this cluster influence (8.77 µg m^−3^). A further 20.8% (C2) and 20.4% (C4) of the trajectories were sourced from Taiwan and the Pearl River Delta (PRD), respectively, yielding PM_2.5_ levels of 7.41 and 8.14 µg m^−3^. The remaining 29.4% of the trajectories were advected from the coastal Philippines, with a corresponding PM_2.5_ concentration of 8.00 µg m^−3^. Notably, dust sources constituted the dominant fraction of spring aerosols, accounting for >30% of the total trajectories and even exceeding 40%. The enhanced contribution of fugitive dust can be attributed to industrial and construction, as well as the long-range dust transport [43,44]. All four clusters traverse industrially developed regions; consequently, dust advected from these source areas exerts a substantial influence on the aerosol burden over Hainan.

In summer (Figure 9b), regional transport is overwhelmingly oceanic: all trajectories originate from the seas south of Hainan. The corresponding marine contribution to aerosol mass is markedly higher than in other seasons, and surface PM_2.5_ reaches its annual minimum. The pronounced dilution by clean maritime air masses is a primary driver of the improved ambient air quality observed over Hainan during summer.

In autumn (Figure 9c), the clusters C3 and C4, which originated from the YRD and Fujian Province, collectively accounted for 67.6% of the total trajectories and yielded the highest PM_2.5_ concentrations of 9.00 and 9.39 µg m^−3^, respectively. Relative to other clusters, these two clusters were characterized by a pronounced enhancement in combustion-related emissions, contributing 51.0% and 54.9% of the total aerosol mass. This increase is likely attributable to industrial activities in the YRD and Fujian regions, as well as to post-harvest biomass burning during the autumn season.

In winter (Figure 9d), all clusters are from the northeast, with typical winter airflow characteristics. The most obvious feature is the increase in secondary sources, accounting for more than 30%, indicating that the clusters in winter are significantly affected by anthropogenic influences. However, Hainan has a sparse distribution of industries, accompanied by a large number of pollutants carried by air masses gradually moving southward in the cold air, which is one of the main reasons for the increase in pollutant concentrations in Hainan in winter. Combining the trajectories of the four seasons, it can be found that the trajectories from the YRD, PRD, and Fujian Province carry larger concentrations of pollutants, while the marine air masses from the south are cleaner.

## 4. Conclusions

In regions with minimal local emissions, regional transport is a significant pathway contributing to the elevation of pollutant concentrations; determining and quantitatively evaluating the composition and sources of water-soluble ions in PM_2.5_ plays a significant role in the development of policies aimed at controlling particulate pollution. We have generated a high-quality dataset for PM_2.5_ composition, derived from the continuous monitoring of chemical constituents at a regional background site in Hainan from August 2021 to July 2022. The results from the Positive Matrix Factorization (PMF) and backward trajectory analysis were utilized to elucidate the chemical characteristics and assess the regional transport patterns of the identified sources.

During the monitoring period, the average mass of total water-soluble ions at the regional station was 7.0 ± 4.4 µg m^−3^, constituting 73.5% ± 24.4% of PM_2.5_ mass. The concentration hierarchy was dominated by secondary inorganic aerosols (SNA), particularly SO_4_^2−^, NO_3_^−^, and NH_4_^+^, indicating significant secondary pollution. The sharp increase in NH_4_^+^ and NO_3_^−^ concentrations with rising PM_2.5_ levels highlights their role in pollution formation. SO_4_^2−^, despite low SO_2_ levels and no coal heating in winter, remained a major component, suggesting external source contributions and a possible link to marine-derived dimethyl sulfide (DMS). The contribution of identified sources, including secondary sources (43.6%), combustion sources (33.9%), dust sources (11.7%), and oceanic sources (10.8%), had different spatial distributions and seasonal variations. Land-based air masses originating from the YRD, PRD, and Fujian are the dominant pathway driving PM_2.5_ elevations over Hainan, whereas southerly marine air masses consistently supply clean background conditions. Specifically, long-range dust and secondary aerosols prevail in winter–spring, combustion emissions peak in autumn, and the summer minimum in PM_2.5_ is governed by marine dilution—seasonal modulation of regional transport thereby governs Hainan’s air-quality evolution. This study offers a scientific foundation for the formulation of more robust air pollution prevention and control measures for Hainan Province and China in the future.

## Figures and Tables

**Table 1:** Summary of linear correlation between the measured and predicted values.

Species	Category	R^2^	Intercept	Intercept SE	Slope	Slope SE	SE
Cl^−^	Strong	0.986	0.017	0.004	0.954	0.006	0.038
NO_3_^−^	Strong	0.438	0.636	0.038	0.295	0.019	0.440
SO_4_^2−^	Strong	0.502	0.675	0.119	0.609	0.034	1.047
Na^+^	Strong	0.994	−0.005	0.002	1.019	0.005	0.017
K^+^	Strong	0.990	0.012	0.001	0.933	0.005	0.015
NH_4_^+^	Strong	0.610	0.322	0.045	0.569	0.026	0.455
Mg^2+^	Strong	0.851	0.011	0.001	0.810	0.019	0.014
Ca^2+^	Strong	0.994	0.000	0.001	0.997	0.004	0.006

**Table 2:** Concentration of PM_2.5_ and water-soluble inorganic ion components (µg m^−3^).

Season	Spring	Summer	Autumn	Winter	Mean
SO_4_^2−^	3.3 ± 1.7	1.7 ± 0.8	2.9 ± 1.7	3.9 ± 1.9	2.9 ± 1.8
NO^3−^	1.4 ± 0.7	0.79 ± 0.2	1.1 ± 0.6	2.8 ± 2.2	1.5 ± 1.4
NH^4+^	1.4 ± 0.7	0.73 ± 0.34	1.2 ± 0.7	2.2 ± 0.75	1.4 ± 1.1
Cl^−^	0.56 ± 0.31	0.32 ± 0.16	0.44 ± 0.31	0.70 ± 0.41	0.50 ± 0.34
Na^+^	0.32 ± 0.21	0.19 ± 0.10	0.29 ± 0.22	0.31 ± 0.23	0.28 ± 0.20
K^+^	0.19 ± 0.21	0.11 ± 0.07	0.15 ± 0.15	0.29 ± 0.14	0.18 ± 0.16
Ca^2+^	0.15 ± 0.086	0.10 ± 0.056	0.052 ± 0.047	0.11 ± 0.081	0.10 ± 0.077
Mg^2+^	0.065 ± 0.043	0.032 ± 0.028	0.041 ± 0.039	0.058 ± 0.049	0.049 ± 0.042
SNA	6.1 ± 3.2	3.2 ± 1.3	5.3 ± 3.3	9.0 ± 5.5	5.9 ± 4.0
TWSI	7.4 ± 3.7	3.9 ± 1.3	6.3 ± 3.3	10.4 ± 5.5	7.0 ± 4.4
PM_2.5_	9.0 ± 2.9	6.9 ± 1.1	9.2 ± 4.1	13.0 ± 5.8	9.5 ± 4.4

## Data Availability

All data supporting this article have been included as part of the main text.

## Abbreviations

The following abbreviations are used in this manuscript:

PMF	Positive Matrix Factorization
WSI	Water-soluble Ions
SNA	Sulfate (SO_4_^2−^), Nitrate (NO^3−^), and Ammonium (NH_4_^+^)
EPA	Environmental Protection Agency
TWSI	Total Water-soluble Ions
DMS	Dimethylsulfide
SOR	Sulfur Oxidation Rate
NOR	Nitrogen Oxidation Rate
